# Enhancing cancer therapy: the integration of oncolytic virus therapy with diverse treatments

**DOI:** 10.1186/s12935-024-03424-z

**Published:** 2024-07-11

**Authors:** Zhuo Yan, Zhengbo Zhang, Yanan Chen, Jianghua Xu, Jilong Wang, Zhangquan Wang

**Affiliations:** 1 Department of Clinical Medical Laboratory Center, Tiantai People’s Hospital of Zhejiang Province (Tiantai Branch of Zhejiang Provincial People’s Hospital), Hangzhou Medical College, Taizhou, 317200 Zhejiang China; 2 Joint Centre of Translational Medicine, Wenzhou Institute, University of Chinese Academy of Sciences, Wenzhou, 325000 Zhejiang China

**Keywords:** Cancer, Oncolytic virus, Immunotherapy, Combination therapy

## Abstract

As one of the significant challenges to human health, cancer has long been a focal point in medical treatment. With ongoing advancements in the field of medicine, numerous methodologies for cancer therapy have emerged, among which oncolytic virus therapy has gained considerable attention. However, oncolytic viruses still exhibit limitations. Combining them with various therapies can further enhance the efficacy of cancer treatment, offering renewed hope for patients. In recent research, scientists have recognized the promising prospect of amalgamating oncolytic virus therapy with diverse treatments, potentially surmounting the restrictions of singular approaches. The central concept of this combined therapy revolves around leveraging oncolytic virus to incite localized tumor inflammation, augmenting the immune response for immunotherapeutic efficacy. Through this approach, the patient's immune system can better recognize and eliminate cancer cells, simultaneously reducing tumor evasion mechanisms against the immune system. This review delves deeply into the latest research progress concerning the integration of oncolytic virus with diverse treatments and its role in various types of cancer therapy. We aim to analyze the mechanisms, advantages, potential challenges, and future research directions of this combination therapy. By extensively exploring this field, we aim to instill renewed hope in the fight against cancer.

## Introduction

Cancer is becoming a leading cause of death globally. From the peak in 1991 to the recent year 2020, the cancer death rates of males and females have decreased by 33%. The reduction in the number of cancer deaths has prevented approximately 3.8 million deaths [1]. The conventional therapies for cancer, such as surgery, chemotherapy, radiotherapy, targeted therapies, or hormonal therapies, deliver limitedly sustained efficacy for the great majority of advanced cancer patients [2].

How does cancer develop? During the process of differentiation, living cells within complex organisms gradually adopt phenotypic states of progressive specificity. Cancerous cells and tissues violate this property, exhibiting increased plasticity of cell states, tissue structure, and function during their progression [3]. However, the immune system closely interacts with tumors throughout the entire process of disease development and progression to metastasis. This complex communication between the immune system and cancer cells can both inhibit and promote tumor growth, and is currently classified as a hallmark of cancer. These interactions, occurring among and across the characteristics of cancer, determine the ultimate outcome [4]. After immune checkpoint inhibitors, oncolytic viruses (OVs) have become the rising stars.

As one of the therapy of immunotherapy, viruses have been explored as potential agents for cancer treatment for over a century [5]. Oncolytic virotherapy is an innovative form of immunotherapy that utilizes natural or genetically modified viruses to selectively infect and destroy cancer cells, while sparing normal cells from harm. In the 18 century, the Bohemian anatomist Vaclav Trnka and the French surgeon Henri François Le Dran described the regression after a tertian malaria with fever and infection with severe inflammation and gangrene of the tumor site [6]. In 1986, a 42 year-old female with acute leukemia experienced temporary remission after infection [7]. Meanwhile, since the mid-1800s, there have been continuous case reports documenting instances of tumor regression occurring concurrently with natural viral infections [5]. However, due to limited medical conditions, technology, and understanding of the mechanisms of viruses and tumors at that time, viruses did not completely revolutionize cancer treatment, and the development of OVs gradually stagnated. With the progress of modern genetic engineering and viral gene research, the use of genetic engineering to design and manipulate viruses has become a promising approach for OVs. In 1991, Martuza, R. L., et al. reported the use of a herpes simplex virus-1 (dlsptk) to inhibit glioma in mice, marking a landmark event in the use of genetic engineering to modify OVs [8]. And OVs then began to develop rapidly. In 1996, research reported the prospect of utilizing mutant adenoviruses to treat specific human tumors [9]. And in 1997, an E1B gene-attenuated adenovirus, ONYX-015, can induce tumor-specific cell lysis and anti-tumor effects [10]. In 2004, RIGVIR, the first OV, received approval for the treatment of melanoma in Latvia [11], followed by H101 in China in 2005 [12], and T-VEC in the United States and Europe in 2015 [13]. The approval of T-VEC attracted increasing attention to oncolytic virotherapy, which marked the maturity of OVs technology and the recognition of its effectiveness. In 2017, Cell reports combination therapy of T-VEC with Keytruda for melanoma treatment, which sparked a research boom in combination therapy involving OVs [14]. In 2021, Deltyact (Teserpatricev/G47 Δ), a modified HSV, received regulatory approval and entered the market in Japan [15]. To date, numerous genetically engineered OVs have undergone various clinical trials, yielding significant results, positioning OVs as one of the most promising immunotherapeutic approaches for cancer in clinical practice.

In this review, we thoroughly explore the recent advancements in research regarding the combination of OVs with various treatments and their role in diverse types of cancer therapy. Our objective is to examine the mechanisms, advantages, potential challenges, and future research directions of this combination therapy. Through an in-depth exploration of this field, our goal is to inspire renewed optimism in the battle against cancer.

## The role of OVs in *Cancer* immunotherapy

To date, there are many OVs have entered into early-phase clinical trials, such as adenoviruses, herpes viruses, measles viruses, coxsackie viruses, polioviruses, reoviruses, poxviruses and Newcastle disease viruses, among others [16] (Table 1). When OVs are employed in cancer therapy, they exhibit a series of intricate and precise mechanisms of action.

**Table 1:** Features of select OVs

	Adenovirus [16, 17]	Vaccinia virus [18]	Herpes virus [19]	Reovirus [20]	Newcastle disease virus [21]	Measles virus [17]
Genome	dsDNA	dsDNA	dsDNA	dsRNA	ssRNA	ssRNA
Genome size	Moderate (35 kb)	Large (194 kb)	Large (154 kb)	Small (23 kb)	Small (15 kb)	Small (11 kb)
Diameter	70–90 nm	360*270*250 nm	150–200 nm	60–80 nm	100–500 nm	100–200 nm
Shape	Spherical	Spheroidicity	Spherical	Spherical	Spherical	Spherical
Cell entry mechanism	Endocytosis	Macropinocytosis	Membrane penetration and fusion	Endocytosis	Endocytosis; pH-independent direct fusion	Membrane fusion
Cell receptors	CAR	GAGs; EFC	HVEM; nectin 1; nectin 2	JAM-A; α-SA	Neuraminidase receptor	CD46; SLAM
Transgene capacity	Moderate	High	High	Moderate	Low	Low
Replication site	Nucleus and cytoplasm	Cytoplasm	Nucleus and cytoplasm	Cytoplasm	Cytoplasm	Cytoplasm
Immunogenicity	Low	High	Low	Low	Low	Low
Blood–brain barrier penetration	Limited	Limited	Limited	Limited	High	Limited

### OVs directly lyse tumor cells

OVs possess a high selectivity for infecting tumor cells. They preferentially infect and destroy cancer cells without causing harm to normal cells and tissues. There are a variety of ways in which different OVs are able to infect cells. Adenovirus utilizes proteins such as coxsackievirus-adenovirus receptor (CAR), integrins, DSG2 or Cluster of Differentiation (CD) 46 as receptors to invade host cells [22]. Upon binding to these receptors, adenovirus enters the host cell either by membrane fusion or other intracellular pathways. Measles virus also employs CD46 as a receptor for cell entry. Herpes Simplex Virus utilizes receptors such as NECTIN or Herpesvirus Entry Mediator (HVEM) for entry into host cells [23]. Besides, Vaccinia Virus and Newcastle Disease Virus lack specific attachment receptors and enters host cells primarily through the process of endocytosis, utilizing cellular internalization mechanisms [24, 25]. Different OVs utilize various cellular receptors or internalization pathways to invade host cells, which contributes to their specificity and effectiveness in targeting tumor cells while minimizing harm to healthy cells.

**Fig. 1:**
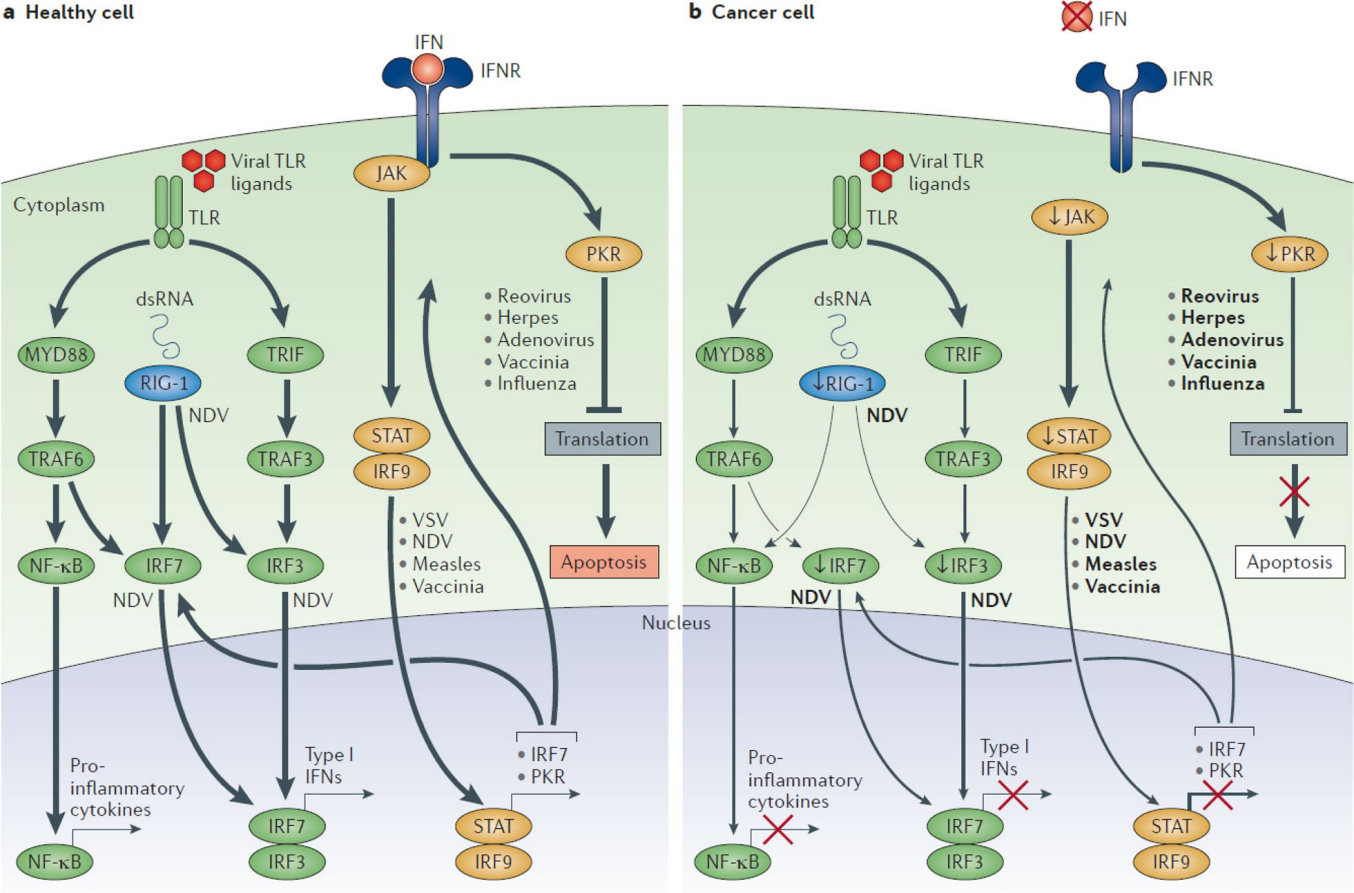
The immune pathways of OVs in healthy cells and cancer cells.** a** Following viral infection, healthy cells activate an antiviral pathway through TLRs and RIG-1, triggering a cascade involving type I IFNs and pro-inflammatory cytokines, which upregulate cell cycle regulators to limit viral spread and promote apoptosis of infected cells.** b** In cancer cells, this process is disrupted by downregulation of key components like RIG-1, IRF7, and IRF3, making them more susceptible to viral replication. This image is modified from that in Kaufman, H. L. et al., Nat Rev Drug Discov, 2015 [16]

Once inside cancer cells, OVs commence replication using the biological machinery of the host cell. This results in extensive viral replication within the cancer cells, ultimately leading to their demise [26]. In the majority of normal cells, after viral infection, they will activate antiviral pathways, thereby inhibiting viral infection. Once detecting a virus, a signaling cascade is initiated through several type I interferon (IFN) elements, including Janus kinase (JAK), signal transducer and activator of transcription (STAT), and interferon regulatory factor 9 (IRF9). This cascade results in a programmed transcriptional pathway that limits viral spread and can target infected cells for apoptosis or necrosis. Local IFN production induced by the innate immune response to viral infections can also enhance antiviral activity through the IFN receptor (IFNR). Toll-like receptors (TLRs) signal through various proteins like myeloid differentiation primary response protein MYD88, TIR-domain-containing adapter-inducing IFNβ (TRIF), IRF7, IRF3, and nuclear factor-κB (NF-κB). This induces the production of pro-inflammatory cytokines and type I IFN. Type I IFN, in turn, signal through the JAK–STAT signaling pathway, leading to the upregulation of cell cycle regulators such as protein kinase R (PKR) and IRF7. These regulators limit viral spread by binding to viral particles and triggering type I IFN transcriptional pathways. This process promotes abortive apoptosis of infected cells and the production of cytokines that alert the immune system to the presence of a viral infection [16, 27]. However, in cancer cells, these signaling pathways might be disrupted, hindering the clearance of viruses. Cancer cells may downregulate key signaling components within the innate signaling pathway, including RIG-1, IRF7, and IRF3. This limits the detection of viral particles by TLR and RIG-1, making cancer cells more susceptible to viral replication. Furthermore, cancer cells may downregulate key components of the type I IFN signaling pathway, thereby limiting the pro-apoptotic and cell cycle regulatory effects of type I IFNs [16] (Fig. 1). When cancer cells dissolve and perish, the viruses are released, continuing to infect and destroy more cancer cells.

### OVs activate immune responses

Absolutely, the activation of both systemic innate and tumor-specific adaptive immune responses is crucial for effectively eliminating tumors using OVs. After oncolytic cell death occurs due to the action of OVs, tumor cells release specific antigens that are associated with the tumor. OVs can activate various forms of immunogenic cell death (ICD) [28], including apoptosis, necroptosis, and pyroptosis. Inducers of ICD are characterized by their ability to stimulate the release of damage-associated molecular patterns (DAMPs) from dying host cells [29, 30]. DAMPs can be classified as constitutive DAMPs (cDAMPs) and inducible DAMPs (iDAMPs). cDAMPs are immune-stimulatory molecules that are continuously expressed before cell death and released by dying cells. iDAMPs are endogenous molecules produced within dying cells as a result of specific cell death pathways [30, 31]. DAMPs include nucleic acids such as mtDNA, ATP, high mobility group box 1 protein (HMGB1), heat shock protein and cytokines such as type I IFNs and IL-1 family [27, 32].

**Fig. 2:**
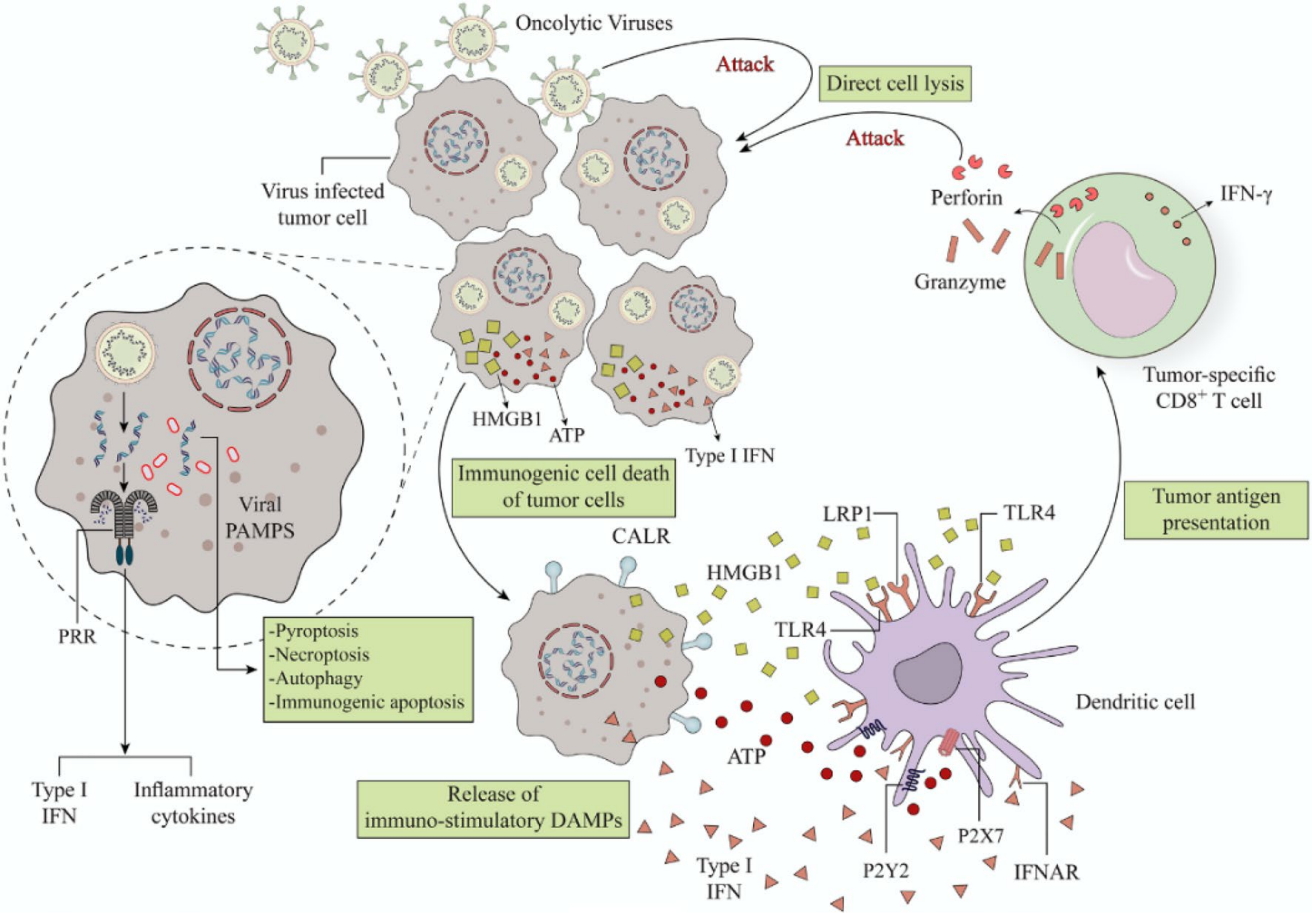
Induction of ICD through OVs and initiation of anti-tumor specific responses facilitated by antigen-presenting cells. This image is modified from that in Mardi, A. et al. Cancer Cell, 2022 [35]

These DAMPs and PAMPs are sensed by pattern recognition receptors (PRRs) [33], such as stimulator of IFN genes (STING), Toll-like receptor (TLR) adaptor molecule 1 and TLR3 on immune cells. This involves creating a proinflammatory environment by triggering the generation of proinflammatory cytokines like type I interferons, tumor necrosis factor α (TNF-α) and interleukin-12 (IL-12). These cytokines will promote the maturation of antigen-presenting cells (APCs). The processed tumor antigens are presented by APCs on their major histocompatibility complex (MHC) molecules, particularly MHC class I molecules. These MHC-peptide complexes are then displayed on the surface of APCs. APCs migrate to secondary lymphoid organs such as lymph nodes, where they interact with T cells. The presentation of tumor antigens by APCs to T cells leads to the activation and expansion of tumor-specific cytotoxic T lymphocytes (CTLs). Activated CTLs migrate to the tumor site guided by chemokines and recognize tumor cells presenting the same antigen they were primed against [34]. They then release cytotoxic molecules such as perforin and granzymes, leading to the destruction of tumor cells [35] (Fig. 2).

**Fig. 3:**
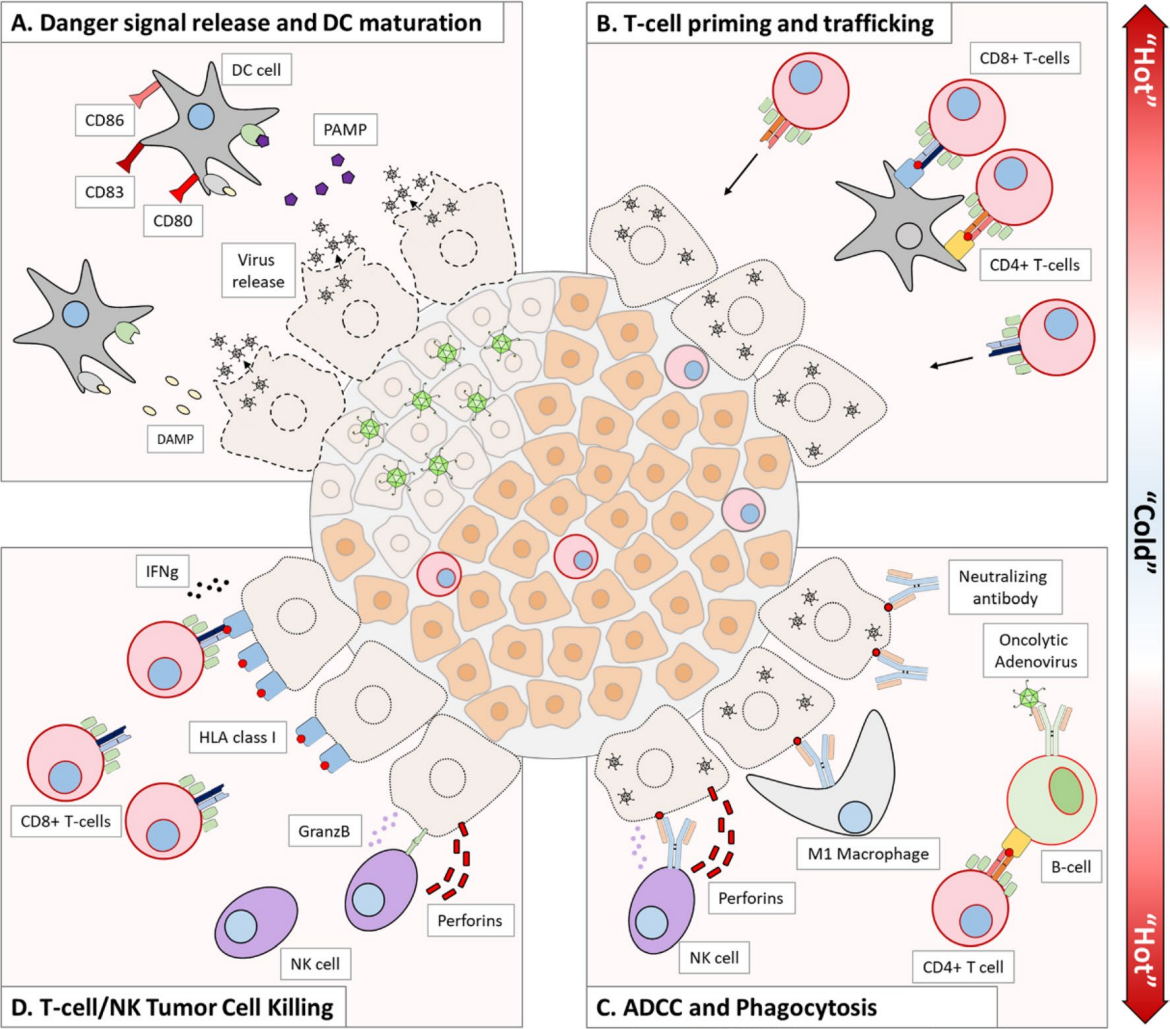
OVs therapy works by activating the immune system to fight against cancer.** a** Oncolytic viruses infect and lyse tumor cells, releasing DAMPs and PAMPs, which activate and mature dendritic cells by upregulating co-stimulatory markers like CD80, CD83, and CD86.** b** Mature dendritic cells process tumor debris, presenting antigens to T cells and attracting them to the tumor site due to the ongoing virus infection.** c** B cells are activated by CD4^+^ T cells or BCR-virus interaction, releasing neutralizing antibodies that mark infected tumor cells for ADCC by NK cells or phagocytosis by M1 macrophages.** d** CD8^+^ T cells and NK cells destroy infected and non-infected tumor cells using INFγ/GranzB and GranzB/Perforins. Oncolytic adenovirus infection upregulates class I HLA in tumor cells, increasing their exposure to CD8^+^ T cells and enhancing immunological activity in the tumor microenvironment. This image is modified from that in Hemminki et al., Journal of Hematology & Oncology, 2020 [2]

This transformation aims to convert immunologically "cold" tumors, which typically lack immune cell infiltration and response, into "hot" tumors. These "hot" tumors are characterized by increased immune cell presence and heightened immune activity, potentially making them more susceptible to immune-mediated attacks or therapies [36, 37]. In the context of cellular innate immunity, NK cells play a significant role in both anti-tumor and antiviral responses. Virus-infected cancer cells often down-regulate or reduce the expression of MHC-I molecules, a strategy to evade recognition and attack by cytotoxic T cells (CTLs). However, this alteration makes them ideal targets for NK cells. NK cells possess activating receptors capable of recognizing the decrease or absence of MHC-I molecules on the cell surface [38, 39]. Activated NK cells kill tumors by releasing cytolytic components, triggering FAS − FAS-ligand signaling, and expressing IFN-γ and TNF-α [33, 40]. These cytokines polarize tumor-supportive M2 macrophages towards proinflammatory M1 phenotypes, activate DCs and recruit more immune cells into the tumor microenvironment (TME) that present antigens to T cells. This NK cells and DCs activation further stimulates the production of IFNs, TNF-α, IL-12, IL-6, and chemokines to amplify the initial innate response [27, 33, 34]. The mainstay of adaptive immunity against tumor cells during OVs infection is the tumor-specific T-cell response. The dead tumor cells release tumor special antigens, which are captured by the antigen-presenting cells (APCs), such as DCs. APCs migrate to the lymph nodes and present these antigens to T cells, initiating an adaptive immune response. These activated T cells, especially CD8^+^ T cells, then enter the tumor tissue to seek out and destroy tumor cells containing the same antigens. In addition, OVs also trigger a potent Type I IFN response, stimulating the production of chemokines recruiting T cells, thereby increasing the infiltration of T cells into TME [41, 42] (Fig. 3).

### OVs recondition/modulate the TME

**Fig. 4:**
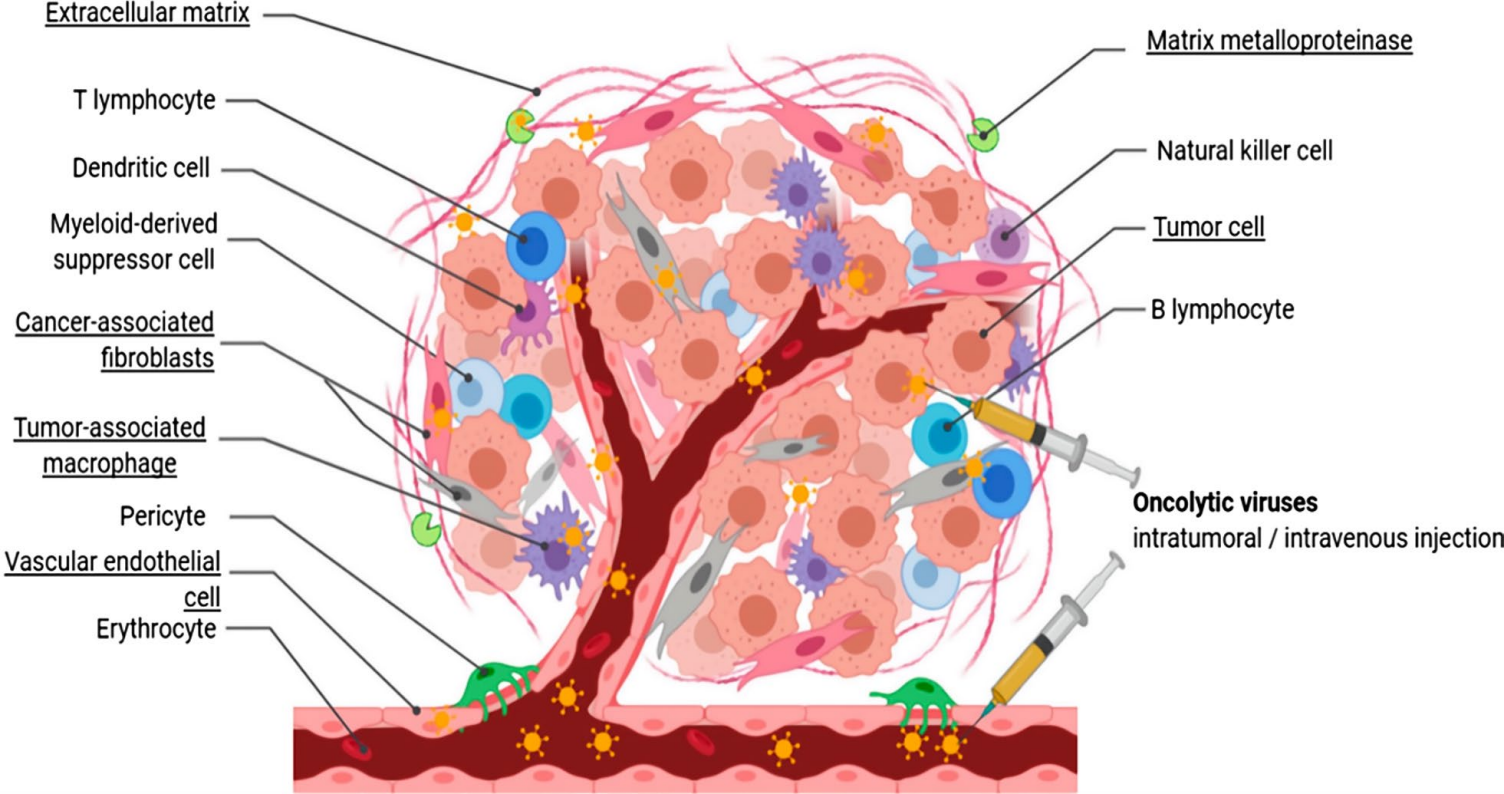
Concurrent targeting of tumors and stroma is achieved through in situ administration of OVs. This includes heterogeneous cancer-associated fibroblasts, extracellular matrix, tumor vasculature, and tumor-associated macrophages. This approach enhances OV penetration and replication, resulting in increased tumor killing and heightened anti-tumoral immune activity. This mange is modified from that in Everts, A. et al., Biomedicines 8, 2020 [45]

When OVs enter the tumor, they also extend to crucial components of the TME. Absolutely, solid tumors are intricate structures that resemble organs in their complexity. They are composed not just of cancerous cells but also include a variety of components such as the extracellular matrix (ECM), vasculature, connective tissue and infiltrating immune cells. ECM is a noncellular compartment, which compromises up to 60% of the tumor mass [43, 44]. The primary source of these ECM molecules are indeed the tumor cells themselves, but notably, cancer-associated fibroblasts (CAFs) contribute to an even greater extent. Therapeutic use of OVs to control the interaction between tumors and stromal components can overcome the therapy resistance and the tumor recurrence [45]. Ilkow et al. demonstrate that the cross-talk between CAFs and cancer cells leads to enhanced growth of OVs therapeutics. Transforming growth factor-b (TGF-b) produced by tumor cells reprogrammed CAFs, dampened their steady-state level of antiviral transcripts and rendered them sensitive to virus infection [46]. Besides, there is study suggesting that in the case of local cell infection, the ratio between fibrous and non-fibrous components of the ECM, as well as the strength of cell-ECM adhesion, significantly impacts the spatial diffusion of tumor cells. Specifically, at extremely high fibrous ratios, there seems to be a tendency for OVs to accumulate at specific spatial locations, resulting in reduced spatial diffusion of tumors under these high fibrous ratio conditions [47] (Fig. 4).

**Fig. 5:**
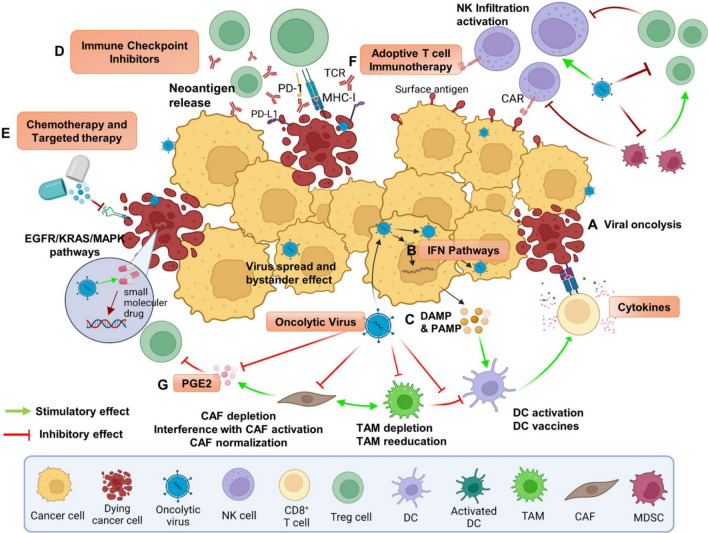
The combination of OVs with immunotherapy and targeted therapies in the TME for cancer.** a** OVs selectively replicate in tumors and directly lyse tumor cells. **b** OVs induce IFN pathways followed by elicitation of immune responses, thus mediating a broader range of long-lasting antitumor effects.** c** OV-mediated increases in the release of DAMPs, PAMPs and cytokines promote the accumulation of CTLs at tumor beds and retention of their killing capability. OVs can synergize with immune checkpoint inhibitors (**d**), chemotherapy and targeted therapy (**e**) to transform the tumor microenvironment from “cold” to “hot” (**g**), thereby improving immune cell recruitment (**f**) and effector function. This image is modified from that in Zhu, Z. et al. Mol Cancer, 2022 [54]

Regarding the vascular system, OVs disrupt tumor vasculature by infecting tumor-associated endothelial and proximal tumor cells, triggering an inflammatory response and the release of TNF-α and IFN-γ [48]. Research indicates that the viral vector VSV possesses a natural ability to target tumor blood vessels, leading to coagulation within these vessels and ultimately resulting in vascular collapse [49]. Hardcastle et al. construct RAMBO (Rapid Antiangiogenesis Mediated by OVs), which expresses Vstat120 under the control of the herpes simplex virus (HSV) IE4/5 promoter. RAMBO treatment induces endothelial cell activation, which inhibits virus propagation and oncolysis in adjacent tumor cells in vitro [50, 51]. Engineered Oncolytic vaccinia virus (OVVs) were demonstrated to specifically target and dismantle existing tumor blood vessels, leading to the destruction of systemic tumors in humans [52]. Interestingly, OVs may also impact neovascularization. VEGI-armed oncolytic adenovirus has been mostly ascribed to its ability to inhibit neovascularization by inducing apoptosis in proliferating endothelial cells [53]. These effects have sparked significant interest among researchers in understanding how We have already made the changes to this. influence the tumor ECM and vascular system (Fig. 5).

## The limitations of OVs monotherapy

Whereas numerous OVs exhibit promising anti-tumor potential in preclinic [55, 56] and clinic [57–59], the common feature that plays a crucial role in prolonging the survival of cancer patients is the induction of specific antitumor immunity, and the efficacy is limited when used as single agents [34, 60, 61].

**Fig. 6:**
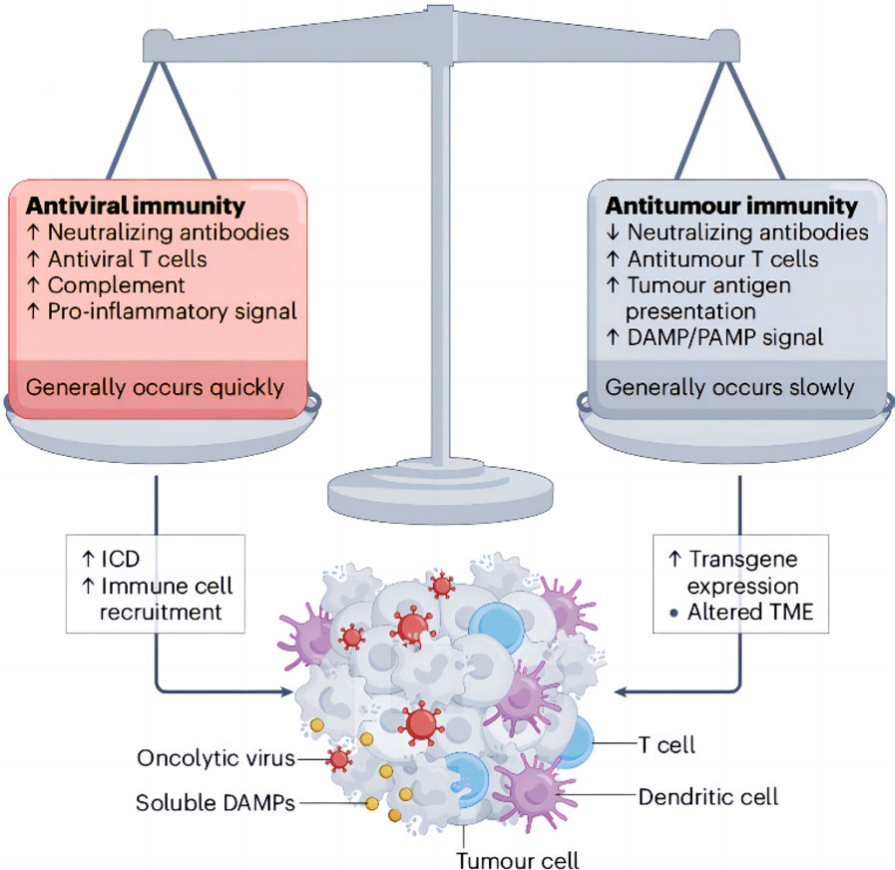
The balance between antiviral immunity and antitumor immunity. The effective rejection of established cancers through immune-mediated processes hinges on maintaining a delicate balance between antiviral immune responses and antitumor immunity. While the immune response against tumors may be enhanced by the immune reactions induced by viral infections, an excessively strong antiviral response can lead to rapid viral clearance, resulting in insufficient activation of antitumor immunity. This image is modified from that in Shalhout, S. Z. et al., Nat Rev Clin Oncol, 2023 [68]

Firstly, OVs may be eliminated by the host immune system prior to reaching the tumor sites. When utilizing OVs for treatment, the neutralizing antiviral antibodies induced by treatment or preexisting antibodies have the potential to impede the ability of OVs to replicate within tumors and induce tumor cell lysis [62]. Besides, complement activation, antiviral cytokines, and macrophages could facilitate the swift elimination of OVs [63, 64]. These antiviral immunities may pose a significant challenge for OVs. Interestingly, pre-existing immunity to Newcastle Disease Virus restricts its replication in tumors, but tumor clearance, abscopal anti-tumor immune effects, and overall survival are not compromised [65] (Fig. 6). Secondly, as OVs must traverse the endothelial layer to reach the tumor site, the extracellular matrix, fibrosis, necrosis, and interstitial hydrostatic pressure consist of the physical barrier, which exert a significant impact on the penetration and diffusion of OVs. The primary impediments to OVs transmission are the ECM, particularly heparan sulfate and collagen [66]. Therefore, adjusting the ECM is essential to enhance the effectiveness of OVs. Thirdly, as only a portion of patients receiving OVs treatment ultimately experience therapeutic benefits, selecting suitable oncolytic virotherapy for patients presents a challenge due to variations in tumor types, stages, and their inherent heterogeneities. Research indicates that identifying clinical biomarkers can assist in predicting a positive response from patients to specific OVs, thereby holding the potential to enhance the outcomes of oncolytic virotherapy [54, 67].

## Current status of oncolytic virotherapy in clinical settings

Oncolytic virotherapy is an emerging and promising approach in cancer treatment that exploits genetically modified viruses to selectively infect and lyse cancer cells, while sparing normal cells and stimulating an anti-tumor immune response. This therapeutic strategy leverages the natural cytotoxic properties of viruses, enhancing them through genetic engineering to improve specificity and efficacy against cancer cells. The therapeutic potential of OVs has been increasingly recognized, leading to significant advancements in both research and clinical applications. Various oncolytic viruses, such as adenoviruses, herpes simplex viruses, reoviruses, and vaccinia viruses, have been engineered and tested for their ability to treat different types of cancer. These viruses have been modified to reduce pathogenicity and enhance tumor specificity, often by inserting genes that encode immune-stimulating factors, thereby turning the tumor into a factory for its own destruction.

In clinical settings, oncolytic virus therapy has shown promise in treating a variety of cancers, including melanoma, glioblastoma, pancreatic cancer, and others. One of the most notable successes is the approval of Talimogene laherparepvec (T-VEC), a genetically modified herpes simplex virus, by the U.S. Food and Drug Administration (FDA) for the treatment of advanced melanoma [13]. T-VEC not only directly lyses tumor cells but also produces granulocyte–macrophage colony-stimulating factor (GM-CSF), which enhances the body's immune response against the tumor. The recognition of the therapeutic potential of OVs has spurred numerous clinical trials worldwide, testing the efficacy and safety of various oncolytic viruses, both as monotherapies and in combination with other cancer treatments such as chemotherapy, radiotherapy, and immune checkpoint inhibitors. These combination therapies aim to exploit the synergistic effects of different treatment modalities, potentially leading to better clinical outcomes for patients.

Despite the significant promise and advancements of oncolytic virotherapy, several challenges and obstacles persist in its clinical application. Effective delivery remains a major hurdle, as systemic administration often leads to rapid clearance by the immune system, and intratumoral injection is not feasible for all tumor types, especially those that are inaccessible or metastatic [69–72]. The heterogeneous and often immunosuppressive tumor microenvironment can further inhibit the spread and replication of OVs [73]. Additionally, pre-existing immunity to common viruses can neutralize OVs before they reach the tumor cells, and the induced immune responses can limit viral replication and spread. Tumors may also develop resistance mechanisms, reducing the efficacy of oncolytic virotherapy over time. Moreover, safety concerns are another significant issue. The immune activation triggered by OVs can sometimes lead to an excessive inflammatory response, known as a cytokine storm, which can be harmful or even fatal [74, 75]. Regulatory and manufacturing challenges also pose significant barriers. Obtaining regulatory approval for new OVs requires extensive safety and efficacy testing, which is both time-consuming and costly. Producing OVs on a large scale while maintaining consistency and quality is another complex challenge, as the manufacturing process must ensure that the viruses are safe, potent, and free of contaminants.

**Fig. 7:**
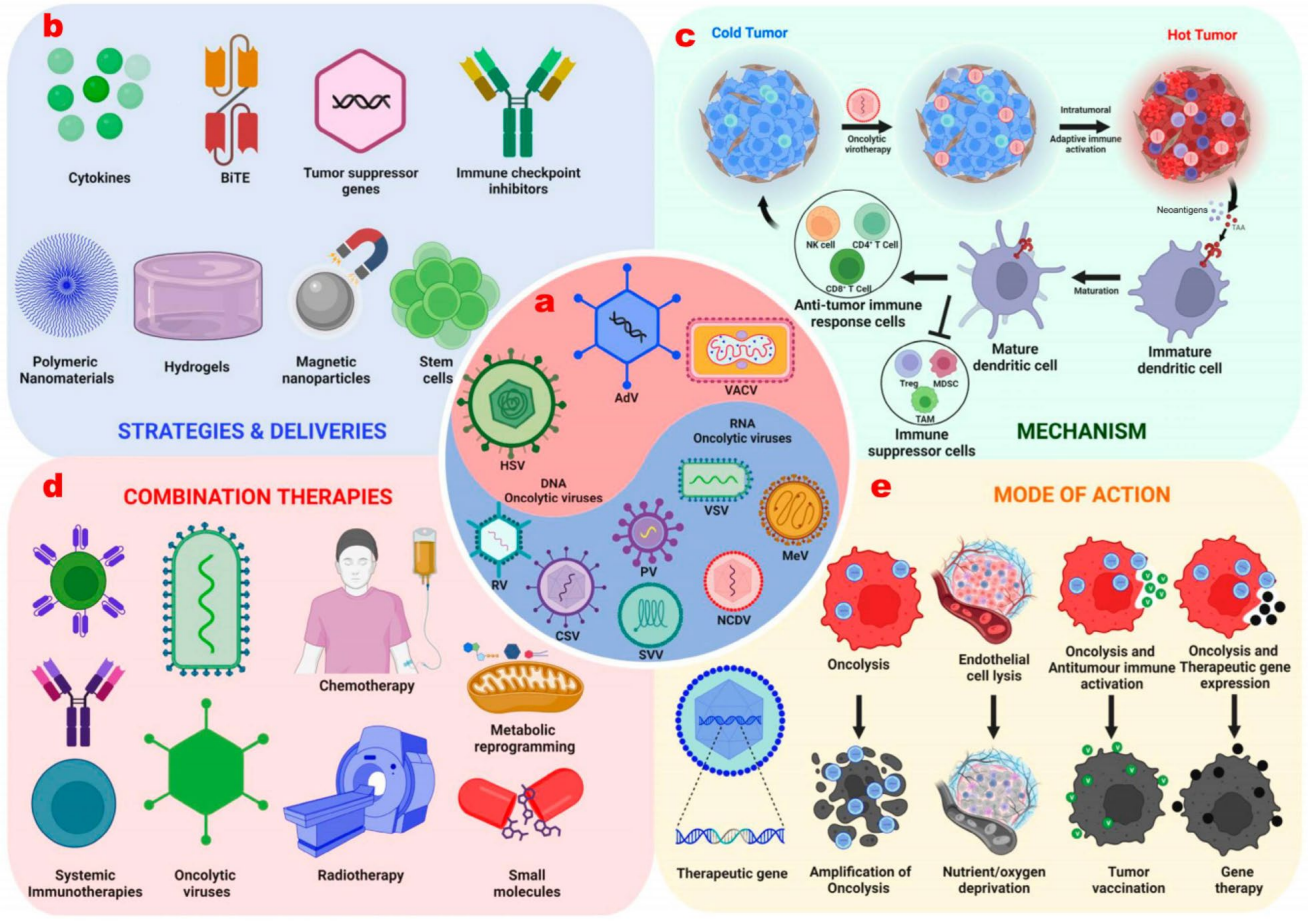
The strategies to enhance the therapeutic efficacy of oncolytic virotherapies. **a** DNA and RNA viruses serve as tools for genetic manipulation, enabling the creation of OVs. **b** Various approaches facilitate OVs reaching tumors. **c** The OVs transform immunologically “Cold” tumors into “Hot” tumors through oncolysis and immunological anti-tumor activities. **d** The combination of OVs with traditional therapies. **e** Genetically engineered OVs cause damage to tumor cells through various means. This image is modified from that in Muthukutty, P. & Yoo, S. Y. Cancer Immunotherapy Perspective, 2023 [83]

One promising direction is the enhancement of oncolytic virus' specificity and efficacy through advanced genetic engineering techniques [76, 77]. This can potentially improve the targeting of cancer cells while minimizing damage to normal cells. Developing novel delivery systems to enhance the stability and delivery efficiency of oncolytic virus is another crucial research area. For instance, employing nanotechnology and biomaterials [69, 72] can help improve the persistence and targeting of viruses in the body, leading to more effective treatments [78, 79]. Additionally, exploring combination therapies that integrate oncolytic virus with immunotherapies, targeted therapies, or radiotherapy could yield synergistic effects, thereby improving overall treatment outcomes. Optimizing dosage and administration regimens through clinical trials is also vital, as it can help maximize therapeutic effects while minimizing adverse side effects [68]. Regulating the tumor microenvironment to enhance the antitumor activity of oncolytic viruses is another important focus. Research into how to activate tumor-infiltrating lymphocytes and increase tumor sensitivity to oncolytic viruses can lead to more effective treatment strategies [80–82] (Fig. 7).

## The combination of oncolytic virotherapy with other treatments

**Fig. 8:**
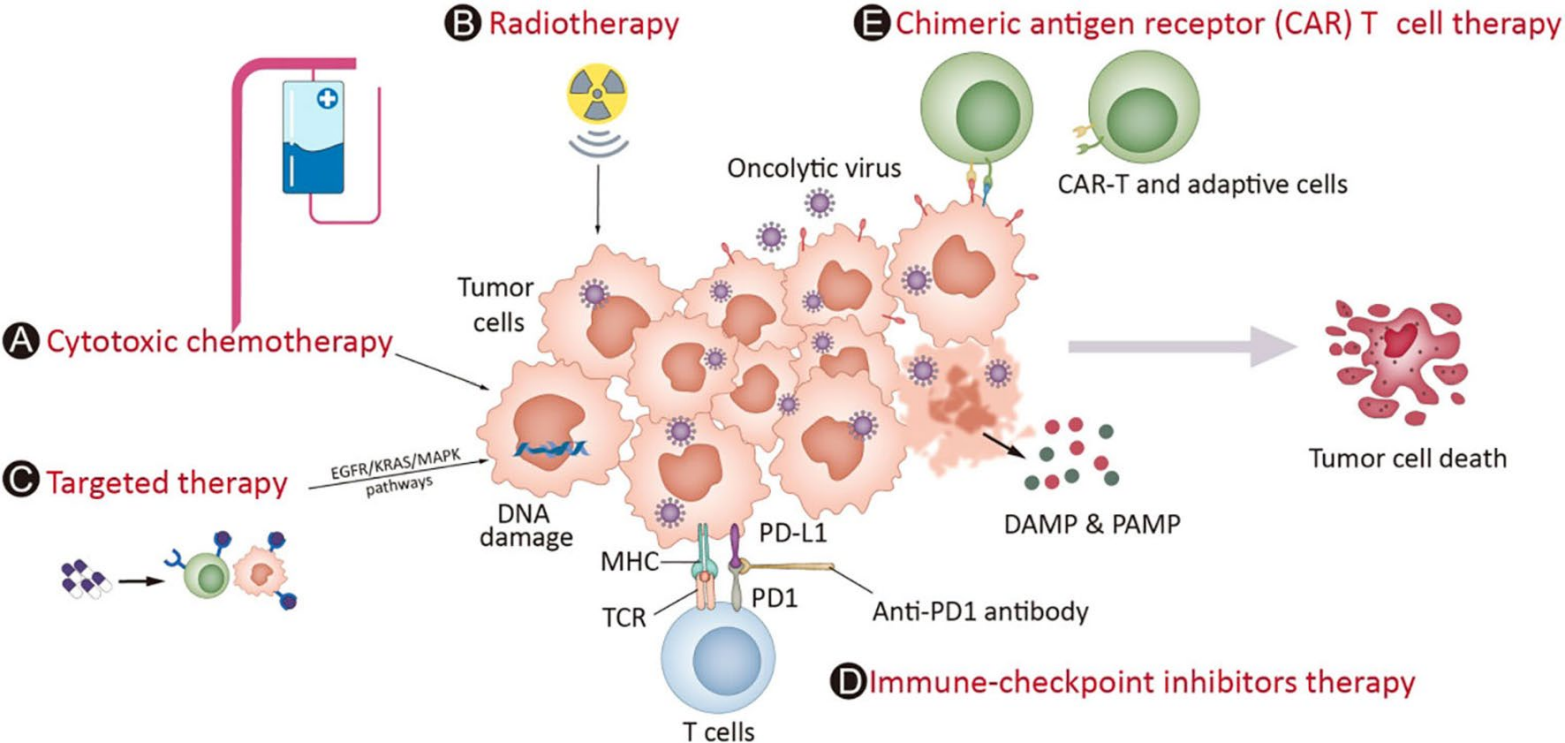
The combination strategies based on OVs in the clinic. OVs directly destroy tumor cells, releasing signals that attract immune cells and enhance their killing abilities. Conventional treatments like chemotherapy (**A**) and radiotherapy (**B**) complement virotherapy. Targeted therapies (**C**) disrupt abnormal signaling pathways, inducing mild immune responses. OVs also upregulate immune checkpoint molecules, creating a more responsive microenvironment, synergizing with immune checkpoint inhibitors (**D**). Combining chimeric antigen receptor (CAR) T cell therapy with OVs enhances T cell penetration into tumors, improving overall efficacy in cancer treatment (**E**). This image is modified from that in Chen, L. et.al, Front Immunol, 2023 [61]

Single-agent therapies are rarely successful in treating cancer, particularly at metastatic or end stages, and survival rates with monotherapies alone are generally poor, influenced by the viral platform and interactions with the host [84]. The combination of multiple therapies to treat cancer has already driven significant improvements in the standard of care treatments for many types of cancers, which can improve the outcomes (Fig. 8).

### The combination of OVs with chemotherapy

Chemotherapy is one of the common treatments for cancer. Cytotoxic agents are the most common drugs used for cancer chemotherapy, which is designed to kill cancer cells resulting in tumor regression and eradication. Most chemotherapy drugs mainly target proliferating cells and exert their effects by inhibiting DNA replication and the cell cycle. Although chemotherapy predominantly affects cancer cells, some proliferating benign cells may also be affected [85, 86]. Almost all the available chemotherapeutic drugs for cancer treatment exert negative effects on rapidly dividing and developing non-cancerous cells. Besides, tumors commonly develop resistance to chemotherapy drugs, often leading to the failure of chemotherapy outcomes [87]. The oncolytic virotherapy uses viruses to selectively target cancer cells, thus minimizing damage to healthy tissues. Interesting, a finding indicates that chemotherapy might lower the required quantity of viral particles to effectively eliminate the tumor [88]. Over the past few years, several studies have demonstrated that the combination of OVs with chemotherapeutic drugs has considerably enhanced efficacy when compared to the initial single-drug treatment. Combining these two therapies might generate a synergistic effect by boosting the sensitivity of cancer cells, enhancing the treatment's effectiveness. The use of Recombinant Adenovirus-based Oncorine (rhAd5), also known as H101, for the treatment of nasopharyngeal carcinoma in combination with chemotherapy was approved in China in 2005 [89]. The combination of low-dose Mitomycin C with oncolytic HSV-1 in a mouse model of dMMR CRC can further enhance tumor sensitivity to immune checkpoint inhibitors [90]. In a phase II clinical trial, patients with stage 2–3 triple-negative breast cancer (TNBC) received five intratumoral injections of T-VEC with paclitaxel followed by doxorubicin and cyclophosphamide, and underwent surgery to assess the Residual Cancer Burden (RCB). The primary endpoint was the RCB0 rate. Secondary end points include RCB0-1 rate, recurrence rates, toxicity, and immune-related factors. The results of the research on TNBC benefits patients from the combination of T-VEC with neoadjuvant chemotherapy (NAC) [91]. In fact, OVs have shown promising synergistic interactions with a wide range of cytotoxic agents.

### The combination of OVs with radiotherapy

Radiotherapy is aim to selectively induce cytotoxic effects on tumor cells within the patients through the strategic application of ionizing radiation [92]. Radiotherapy disrupts cancer cells through direct damage to their DNA or create oxygen radicals that interfere with cellular pathways. OVs combine with radiotherapy, exhibit dual effects. Firstly, OVs can mitigate the DNA damage caused by radiotherapy by sequestering DNA damage repair proteins, essentially functioning as sensitizers to radiotherapy [93, 94]. Simultaneously, radiotherapy induces apoptosis in tumor cells, leading to the release of numerous TAAs and DAMPs, consequently fostering the replication and dissemination of OVs [95]. Furthermore, radiotherapy may augment viral uptake, viral gene expression, and viral replication [96]. Several research studies have shown that the combination of OVs and radiotherapy yields promising therapeutic outcomes that cannot be achieved through monotherapy alone. Recently, the combination of oAd DNX-2401 and radiotherapy has emerged as a promising approach for incorporating immunotherapy into the treatment regimen for patients diagnosed with newly identified diffuse intrinsic pontine glioma (DIPG). Out of the 12 glioma patients who underwent a single intratumoral (IT) infusion of viral particles containing DNX-2401, 11 subsequently underwent radiotherapy, typically at a median dose of 54 Gy. Nine of twelve patients exhibited increased T cell activity, leading to either reduced tumor size or stabilization thereof [97]. Also, one study illustrated that the irradiation of Brain Tumor Initiating Cells (BTICs) amplified the lytic activity of the YB-1-dependent oncolytic adenovirus XVir-N-31 [98].Interesting, researchers are also investigating OVs as potential radiosensitizers, especially those possessing DNA genomes that undergo a portion of their replication cycle within the nucleus of host cells [99]. One research demonstrates that the combination of external beam radiation and EnAd oncolytic virotherapy, which results in a synergistic or at least additive impact on tumor cytotoxicity. This outcome is attributed to the interplay between EnAd and multiple DNA damage response (DDR) pathways, accompanied by an increased production of the virus. Consequently, this process results in heightened cell death through oncosis in irradiated cultures [100].

### The combination of oncolytic virotherapy with targeted therapy

Targeted therapy is a cancer treatment strategy that selectively intervenes in the specific biological processes of tumor cells, inhibiting the growth and spread of cancer cells. Unlike traditional radiotherapy and chemotherapy, targeted therapy is more precise, capable of identifying and attacking specific molecules or pathways in cancer cells, thereby minimizing the impact on normal cells [101, 102]. The mechanisms of targeted therapy are diverse, including antibody therapy, small molecule inhibitors, and more. Antibody therapy involves the use of artificially synthesized antibodies that bind to specific antigens on the surface of cancer cells, triggering the immune system to attack the tumor [103]. Small molecule inhibitors, on the other hand, interfere with signaling pathways [104, 105]or other biological processes within cancer cells, inhibiting their growth and division.

The advantages of targeted therapy lie in its precision, reducing damage to surrounding normal tissues, and enhancing treatment specificity and efficacy. However, specific gene mutations in cancer treatment targets may increase drug sensitivity or more frequently lead to treatment resistance [106]. Therefore, combining targeted therapy with oncolytic virotherapy can further increase treatment specificity and effectiveness, providing a more potent means to eliminate tumor cells and reduce the risk of tumor recurrence. In a phase 1/2 trial, oncolytic DNX-2401 virotherapy was combined with pembrolizumab in recurrent glioblastoma, and Combination therapy results in significant survival benefits for specific patients [107]. In a study, pre-treating malignant peripheral nerve sheath tumors with ruxolitinib, a selective inhibitor targeting JAK-1/2, made the tumors more susceptible to oHSV infection, enhancing viral replication and altering the immune-mediated response induced by oHSV [108]. Interesting, engineered OVs can be used for targeted therapy. A novel oncolytic poxvirus with thymidine kinase (TK) gene deletion, named ΔTK-Armed-VACV, equipped with anti-programmed cell death protein 1 (PD-1) antibody and anti-human tumor necrosis factor receptor superfamily, member 9 (4-1BB) antibody genes, exhibited significant antitumor effects. This suggests that combining oncolytic VACV with one or more immune checkpoint genes could offer promising clinical prospects in oncolytic virotherapy [109]. L. Tian et al. developed an oncolytic herpes simplex virus (oHSV) type 1 expressing a secreted single-chain variable fragment (scFv) derived from the epidermal growth factor receptor (EGFR) antibody cetuximab, linked to CCL5 using an Fc knob-into-hole strategy to generate heterodimers (OV-Cmab-CCL5). Infecting glioblastoma multiforme (GBM) with OV-Cmab-CCL5 significantly enhances the migration and activation of NK cells, macrophages, and T cells; suppresses tumor EGFR signaling; reduces tumor size; and prolongs the survival time of mice carrying GBM [110].

### The combination of OVs with immune checkpoint inhibitors (ICIs)

An ICI can unleash an immune system attack on cancer cells, which is usually suppressed by tumor cells or the tumor microenvironment. The FDA approval of the ICI anti CTLA-4 antibody Yervoy (ipilimumab) in 2011, anti-PD1 antibodies Keytruda (pembrolizumab) in 2014, and Opdivo (nivolumab) in 2015 are the major milestones of immunotherapy, marking a beginning of new era of cancer therapy. Later, the anti-PD1 antibody Libtayo (cemiplimab) and anti-PD-L1 antibodies Tecentriq (atezolizumab), Bavencio (avelumab), and Imfinzi (durvalumab) have been approved by the FDA [111]. ICIs mainly include PD-1/PD-L1 inhibitors and CTLA-4 inhibitors.

PD-1 is a protein found on the surface of immune cells, and when it binds to its ligand PD-L1, it inhibits the activity of immune cells such as T cells, weakening their ability to attack tumor cells. PD-L1 is typically expressed by tumor cells and certain immune cells, facilitating the evasion of tumor cells from the immune system. The mechanism of action of PD-1/PD-L1 inhibitors involves blocking the binding between PD-1 and PD-L1, thus preventing tumor cells from evading the immune system through this mechanism. This blockade enables immune cells to regain the ability to attack tumor cells, enhancing the immune system's recognition and destruction of cancer cells [112]. Some PD-1/PD-L1 inhibitors include Pembrolizumab (Keytruda), Nivolumab (Opdivo), Atezolizumab (Tecentriq), Avelumab (Bavencio), Durvalumab (Imfinzi), widely used in treating advanced melanoma, non-small cell lung cancer, renal cell carcinoma, bladder cancer and Hodgkin lymphoma, amongst other tumor types [113]. A study found that oncolytic vaccinia virus attracts effector T cells and induces PD-L1 expression in both cancer cells and immune cells within tumors. Combining oncolytic virus therapy with anti-PD-1/PD-L1 therapy not only reduces the number of PD-L1-positive cells but also promotes infiltration of effector CD8^+^ and CD4^+^ T cells into tumor tissues, along with increased expression of IFN-γ, ICOS, granzyme B, and perforin [18]. One research conducted a preclinical investigation, combining the oncolytic herpes simplex virus HSV1716 with an anti-PD-1 antibody for the treatment of murine models exhibiting rhabdomyosarcoma. In this study, mice bearing tumors and treated with the combined therapy displayed significantly prolonged survival compared to untreated mice or those receiving either therapy alone. Notably, favorable treatment outcomes were associated with heightened infiltration of CD4^+^ and CD8^+^ T cells within the tumors, while no notable increases in immunosuppressive Foxp3 + T-regulatory (Treg) cells were observed [114]. Similarly, the oncolytic virus oHSV2, in combination with PD-1/PD-L1 inhibitors, demonstrates potent antitumor activity by facilitating the infiltration of CD4^+^T and CD8^+^T cells within the lymphoma tumor microenvironment. The antitumor effect suggested that the combination therapy of oHSV2 and PD-L1 would have a better prospect for clinical application [115]. Besides, one research reported tumor reduction and prolonged survival of a patient with skin cancer treated with the triple combination OV, radiotherapy and ICI [116]. Interestingly, in a study, researcher generated an engineered OVs that coexpressed a PD-L1 inhibitor and GM-CSF. This engineered OVs are able to activate tumor neoantigen-specific T cell responses, providing a potent, individual tumor-specific oncolytic immunotherapy for cancer patients, especially those resistant to PD-1/PD-L1 blockade therapy [117].

Cytotoxic T-lymphocyte-associated protein 4 (CTLA-4) is an inhibitory receptor belonging to the CD28 immunoglobulin subfamily, expressed primarily by T-cells. CTLA-4 inhibitors block the CTLA-4 on immune cells, revving up the immune response against cancer. When CTLA-4 binds to its ligands (such as CD80 and CD86) on antigen-presenting cells, it inhibits the activation and proliferation of T cells, acting as a negative regulator of immune responses. CTLA-4 inhibitors unleash the ability of immune system to attack tumors by stopping CTLA-4 from putting the brakes on T cell activity [118]. Ipilimumab (Yervoy) is a prominent CTLA-4 inhibitor used in the treatment of advanced or unresectable melanoma, often combined with other therapies to enhance immune responses against cancer cells [119]. Saha et al. demonstrate that a combined treatment involving an oncolytic virus expressing IL-12, coupled with two immune checkpoint inhibitors (anti-CTLA-4 and anti-PD1 antibodies), exhibits the potential to eradicate glioma in two distinct mouse models. The success of this combined therapeutic approach is contingent upon the involvement and interplay of CD4^+^ and CD8^+^ T cells alongside macrophages, showcasing their pivotal role in achieving therapeutic efficacy [120]. Oncolytic virotherapy have the potential to boost the immunogenicity of tumors while reshaping the typically immunosuppressive tumor microenvironment. This transformation can result in heightened antitumor responses to immune-checkpoint inhibitors. One research investigated the therapeutic potential of G47Δ, a third-generation oHSV type 1, in combination with immune-checkpoint inhibitors using various syngeneic murine subcutaneous tumor models. The combination of intratumoral G47Δ with systemic anti-CTLA-4 antibody demonstrated effective recruitment of effector T cells into the tumor while reducing regulatory T cell presence. Additionally, this combination therapy significantly upregulated various gene signatures associated with inflammation, lymphoid lineage, and the activation of T cells. These findings indicate a potential shift from immune-insensitive tumors to a state of increased immune susceptibility [121]. Yu J. L. et al. propose a mathematical model to explore the interactions of combined therapy of OVs and a checkpoint inhibitor, anti-CTLA-4. This model describes the destructive ability of cytokine on tumor cells as well as the inhibitory capacity of CTLA-4 on various components. Also, the model is validated through the experimental results. The research reveals that the combined therapy's primary biological function lies in the activation of host anti-tumor immune system responses rather than directly destroying the tumor cells [122].

### The combination of OVs with adoptive cell therapies (ACT)

The use of genetically modified T cells, either utilizing tumor-infiltrating lymphocytes (TIL), or expressing novel T cell receptors (TCR) or chimeric antigen receptors (CAR), in ACT is another strategy to modify the immune system for the recognition and anti-tumor function against cancer cells [123, 124]. The use of other immune cell types, such as natural killer cells, as the basis for cell therapy is also a current area of research.

Tumor-infiltrating lymphocytes (TIL) has emerged as a promising treatment for solid tumors and has demonstrated anti-tumor efficacy in selected patients with melanoma, ovarian cancer, and non-small cell lung cancer (NSCLC). However, the full potential of TI therapy is hindered by insufficient TIL activation, low persistence, and inefficiency in the presentation of new tumor antigens. One study engineered oncolytic HSV-1 to express trimerized OX40L and IL12, and the combination therapy of OV-OX40L/IL12 and TIL achieved tumor regression [125]. Research has found that TIL can serve as carriers to transport OVs into tumors [126]. Similarly, cellular viral therapy can increase the infiltration of TIL [127].

To redirect T cells against tumor cells, T cells can be engineered ex vivo to express cancer antigen-specific TCR, generating products called TCR-engineered T cells (TCR-T) [128]. Vesicular stomatitis virus (VSV) is a virus with potent oncolytic properties, but it suffers from drawbacks such as low systemic delivery efficiency and severe side effects like neurotoxicity. In a study, VSV was loaded onto CD8^+^ T cells. Compared to systemic administration of the naked virus, not only did this approach enhance safety, but it also efficiently delivered the virus to its tumor targets [129]. In a preclinical experiment, TCR transgenic T cells and YB-1-based oncolytic viruotherapy increased the survival rates of tumor-bearing mice [130]. CAR-T cell therapy is a form of immunotherapy that involves modifying the T cells of patients to make them better at recognizing and attacking cancer cells. This treatment begins by collecting the patient's T cells from their blood. Then these T cells are genetically engineered in a laboratory to produce chimeric antigen receptors (CARs) on their surface. CAR-T therapy has rapidly impacted the malignant tumor field and has achieved remarkable effects in recent years as the latest promising adoptive cell therapy [131]. FDA approved the first CAR-T cell therapeutic product (Kymriah, CTL-019 from Novartis) for patients with relapsed / refractory B-cell acute lymphoblastic leukemia (r/r B-ALL) in 2017 [132, 133]. CAR-T therapy offers a potential treatment avenue for Glioblastoma (GBM). However, it is often impeded by poor cell infiltration within the tumor and a highly immunosuppressive TME. Researchers have utilized oncolytic adenoviruses armed with CXCL11 to enhance CAR-T cell infiltration and reprogram the immunosuppressive TME, aiming to bolster its therapeutic efficacy [134]. One study involved combining CAR-T cells designed to target carbonic anhydrase 9 (CA9) with an oncolytic adenovirus carrying CCL5 and IL12 to investigate the collective anti-tumor effects of this combined approach. The result demonstrated that Ad5-ZD55-hCCL5-hIL12 effectively infected and replicated within renal cancer cell lines, resulting in a moderate inhibition of xenografted tumors in nude mice [135]. Another study has demonstrated that combining oncolytic virus therapy with adoptive T-cell therapy, rapamycin, and celecoxib exerts potent anti-tumor effects on brain tumors. This represents a combination approach utilizing multiple therapies in anti-tumor efficacy [136].

NK cells are a vital component of the innate immune system. In comparison to T cells, NK cells provide multifaceted advantages for tumor immunotherapy. CAR-NK cells may serve as a favorable alternative to CAR-T cells, as they do not require HLA compatibility and have limited toxicity. Additionally, CAR-NK cells may be produced on a large scale from various sources [137, 138]. A study engineered NK cells with CCR5 modification, and although the tumor did not completely regress in an in vivo model, the combination therapy with an oncolytic poxvirus expressing CCL5 resulted in an accumulation of more NK cells within the tumor lesion [139]. One research employed a combination of oncolytic measles vaccine virus (MeV) and activated human NK cells (or PBMC). Compared to their respective monotherapies, the combination treatment strategy resulted in enhanced oncolytic effects against A673 and HT1080 cells [140]. There is another research provides evidence for the potential effectiveness of combining oncolytic parainfluenza virus with PM21-NK cell adoptive therapy for lung cancer [141].

## Conclusion

The amalgamation of OVs with various therapeutic modalities has heralded a new era in the field of cancer treatment. This combined approach offers a novel pathway to overcome the limitations of singular treatment methods, paving the way for innovative treatment schemes and personalized therapies. Further research and clinical practice will aid in deepening our understanding of the interactions between OVs and other treatment modalities, fostering the development of more precise and efficient treatment protocols. The prospects  of personalized therapy and collaborative treatment strategies are immensely promising, holding the potential to overcome challenges in cancer treatment, mitigate adverse effects, and enhance therapeutic efficacy. Therefore, the integration of OVs with diverse treatment approaches marks an exhilarating new chapter in the future of cancer therapy, offering patients a more hopeful and promising outlook for treatment.

## Data Availability

Data will be made available on request.

## Abbreviations

OVs: Oncolytic viruses
CAR: Coxsackievirus-adenovirus receptor
CD: Cluster of Differentiation
HVEM: Herpesvirus Entry Mediator
IFN: Interferons
TLRs: Toll-like receptors
PAMPs: Pathogen-associated molecular patterns
JAK: Janus kinase
STAT: Signal transducer and activator of transcription
IRF9: Interferon regulatory factor 9
IFNR: IFN receptor
NF-κB: Nuclear factor-κB
PKR: Protein kinase R
ICD: Immunogenic cell death
DAMPs: Damage-associated molecular patterns
cDAMPs: Constitutive DAMPs
iDAMPs: Inducible DAMPs
HMGB1: High mobility group box 1 protein
PRRs: Pattern recognition receptors
STING: Stimulator of IFN genes
TNF-α: Tumor necrosis factor α
IL-12: Interleukin-12
APCs: Antigen-presenting cells
MHC: Major histocompatibility complex
CTLs: Cytotoxic T lymphocytes
TME: Tumor microenvironment
ADCC: Antibody-dependent cell-mediated cytotoxicity
ECM: Extracellular matrix
CAFs: Cancer-associated fibroblasts
TGF-b: Transforming growth factor-b
HSV: Herpes simplex virus
T-VEC: Talimogene laherparepvec
FDA: Food and Drug Administration
GM-CSF: Granulocyte–macrophage colony-stimulating factor
TNBC: Triple-negative breast cancer
RCB: Residual Cancer Burden
NAC: Neoadjuvant chemotherapy
DIPG: Diffuse intrinsic pontine glioma
IT: Intratumoral
BTICs: Brain tumor initiating cells
DDR: DNA damage response
TK: Thymidine kinase
PD-1: Anti-programmed cell death protein 1
scFv: Secreted single-chain variable fragment
EGFR: Epidermal growth factor receptor
GBM: Glioblastoma multiforme
ICIs: Immune checkpoint inhibitors
Treg: T-regulatory
CTLA-4: Cytotoxic T-lymphocyte-associated protein 4
ACT: Adoptive cell therapies
TIL: Tumor-infiltrating lymphocytes
TCR: T cell receptors
CAR: Chimeric antigen receptors
NSCLC: Non-small cell lung cancer
TCR-T: TCR-engineered T cells
VSV: Vesicular stomatitis virus
GBM: Glioblastoma
CA9: Carbonic anhydrase 9
MeV: Measles vaccine virus
